# HFE Mutations in Neurodegenerative Disease as a Model of Hormesis

**DOI:** 10.3390/ijms25063334

**Published:** 2024-03-15

**Authors:** Savannah L. Marshall Moscon, James R. Connor

**Affiliations:** Department of Neurosurgery, College of Medicine, The Pennsylvania State University, 700 HMC Crescent Road, Hershey, PA 17033, USA; slm6158@psu.edu

**Keywords:** HFE, iron, oxidative stress, hormesis, neuroprotection, neurodegeneration, Alzheimer’s disease, Parkinson’s disease, amyotrophic lateral sclerosis

## Abstract

Common variants in the iron regulatory protein HFE contribute to systematically increased iron levels, yet the effects in the brain are not fully characterized. It is commonly believed that iron dysregulation is a key contributor to neurodegenerative disease due to iron’s ability to catalyze reactive oxygen species production. However, whether HFE variants exacerbate or protect against neurodegeneration has been heavily debated. Some claim that mutated HFE exacerbates oxidative stress and neuroinflammation, thus predisposing carriers to neurodegeneration-linked pathologies. However, H63D HFE has also been shown to slow the progression of multiple neurodegenerative diseases and to protect against environmental toxins that cause neurodegeneration. These conflicting results showcase the need to further understand the contribution of HFE variants to neurodegenerative disease heterogeneity. Data from mouse models consistently demonstrate robust neuroprotection against toxins known to increase the risk of neurodegenerative disease. This may represent an adaptive, or hormetic, response to increased iron, which leaves cells better protected against future stressors. This review describes the current research regarding the contribution of HFE variants to neurodegenerative disease prognosis in the context of a hormetic model. To our knowledge, this is the first time that a hormetic model for neurodegenerative disease has been presented.

## 1. Introduction

Iron is an indispensable element in the brain, playing a pivotal role in neurotransmitter synthesis, myelin synthesis, metabolism, and many other cellular processes [1]. Iron is heterogeneously distributed in the brain, and many regions with the highest iron concentrations are involved in motor function, such as the substantia nigra (SN) [1,2]. Furthermore, iron dysregulation is thought to play a role in the development of age-related disorders, such as neurodegenerative diseases [3]. In fact, aging increases the risk of iron dysregulation and brain iron overload, specifically in regions implicated in neurodegeneration, such as the SN [4,5,6,7,8,9]. However, brain iron levels are also impacted by genetics, sex, disease states, and many other environmental factors [10,11]. Increased iron levels have been found in the brains of patients with Alzheimer’s disease (AD) [12,13,14,15,16], Parkinson’s disease (PD) [4,17,18,19,20,21,22,23], and amyotrophic lateral sclerosis (ALS) [24,25]. Key pathologies present in the brains of neurodegenerative disease patients are oxidative stress, neuroinflammation, mitochondrial dysfunction, iron overload, and protein misfolding and aggregation [26,27,28,29,30,31,32,33,34,35]. Increased brain iron can exacerbate all pathological processes of neurodegenerative diseases [31,36,37,38,39,40]. The extensive role of iron dysregulation in neurodegeneration has resulted in further investigations into the influence of genes regulating iron homeostasis, none of which have a higher mutation rate than the homeostatic iron regulator (*HFE*) gene [41]. The C282Y mutation in the HFE protein is the main genetic cause of hereditary hemochromatosis (HH), but the H63D mutation is the most common mutation in Caucasians, occurring in approximately 15–30% of the Caucasian population [42]. Carriers of HFE mutations have an increased risk of iron accumulation in the brain and show signs of oxidative damage, hence variants may contribute to the neurodegenerative disease process [40].

The role of HFE variants in the pathology of neurodegenerative diseases remains highly debated. There are reports of a negative impact of the H63D HFE mutation, such as in AD patients, where it has been found that H63D HFE carriers have earlier disease onset [43]. Others have refuted any correlation between HFE mutations and AD [44]. In PD patients, no studies have found an altered risk of disease in those with H63D HFE [45,46,47,48,49,50,51,52], but carriers are less vulnerable to developing PD when exposed to environmental toxins associated with PD risk [53]. Furthermore, several studies report an increased frequency of H63D HFE in ALS patients [54,55,56,57,58], while others report no difference in frequency [24,59,60,61,62]. However, ALS patients with H63D HFE have been observed to undergo slower disease progression, possibly explaining the increased prevalence in ALS patients reported by other studies [63]. The disagreement in this field points to a need to analyze the impact of HFE variants in the context of other factors (genetic, environmental, dietary, etc.) that impact iron metabolism. Animal studies of the impact of HFE variants on neurodegenerative pathology have been pivotal due to their ability to mitigate confounding factors that affect iron homeostasis.

Rodent models have been used to shape the current understanding of the mechanisms of iron regulation in HFE mutation carriers. Mice with H67D HFE, the murine homolog of H63D HFE in humans, have increased astrogliosis and microglial proliferation and migration, indicating elevated neuroinflammation [64]. However, H67D HFE mice have been shown to be significantly protected from motor dysfunction, oxidative stress, and mitochondrial dysfunction when exposed to PD-causing toxins [65,66,67]. Moreover, it has been found that H67D HFE causes oxidative stress and neuroinflammation only in younger mice, and these indications of stress subside sometime after 6 months of age when they also have increased expression of nuclear factor erythroid 2-related factor 2 (Nrf2), a transcription factor responsible for promoting antioxidant expression [64]. This is likely evidence of adaptation to the inherently increased iron and associated oxidative stress caused by H67D HFE. A likely mechanism supported by the current data is the activation of antioxidant pathways, which likely remain elevated long term, a concept that is consistent with the phenomenon of hormesis [68]. Thus, to understand and interpret the impact of HFE mutations on neurodegenerative disease, we strongly suggest that the data be viewed through the lens of the common process known as hormesis.

**Figure 1:**
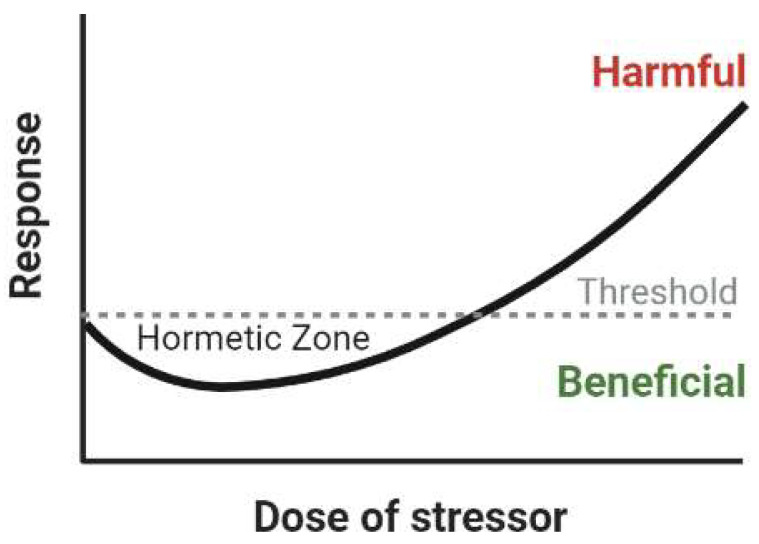
Dose-response curve of a hormetic mechanism. Doses of a toxin below a threshold will have a beneficial impact by causing the upregulation of broad, long-term protective mechanisms. Figure created in Biorender.

Hormesis is defined as a biphasic dose response curve where low doses of a toxin warrant adaptation and long-term protection against broadly related toxins, and only doses above a specific threshold become toxic, as depicted in Figure 1 [68,69,70,71]. Our lab has adopted the hypothesis that the extent to which the brain iron load is increased in those with HFE mutations falls within the hormetic dose of iron and consequently alters the propensity and pathogenesis of neurodegenerative diseases. This review will detail the research in humans and research models with HFE mutations to provide a detailed overview of the potential roles of HFE variants in modifying neurodegenerative disease etiology and provide insight into how this question should be further explored.

## 2. Homeostatic Iron Regulator: HFE

In 1996, a mutation in a novel major histocompatibility complex (MHC) class-I-like gene was found in 83% of patients with hereditary hemochromatosis (HH). The gene was later named *HFE* [72]. *HFE* is categorized as a non-classical MHC class I gene [73]. In contrast to classical MHC class I molecules, which are highly polymorphic, non-classical class I molecules have tissue-specific changes in expression, and relatively few polymorphisms [73]. Studies of tissue distribution of the HFE protein demonstrated its presence in tissues that participate in iron metabolism, yet the gene does not contain an iron response element (IRE) and is not known to be regulated by iron status [74,75]. The overall structure of HFE resembles that of other MHC class I molecules; however, it has a narrow peptide-binding groove, suggesting that it does not present peptides like other MHC molecules [76].

Twenty years after its discovery, it was demonstrated that HFE associates with the transferrin receptor (TfR) [76]. TfR is a homodimeric membrane protein that binds to holo-transferrin (Fe-Tf) [77]. The TfR:Fe-Tf complex is endocytosed into acidic intracellular compartments, where iron dissociates from Tf as a result of the low pH. The TfR remains bound to apo-Tf and is recycled to the cell surface, where apo-Tf is released from the TfR [77]. The HFE protein forms a stable complex with the TfR and causes the receptor to exhibit a lower affinity for holo-Tf by a mechanism of competitive inhibition [77,78,79,80].

### 2.1. HFE Variants and Iron Overload

There are two common variants found in the *HFE* gene: a substitution of tyrosine for cysteine at position 282 (C282Y) and a histidine to aspartic acid at position 63 (H63D). The C282Y variant, or C294Y, in mice prevents correct protein folding and leads to a complete lack of translocation to the cellular membrane [11,73]. Consequently, C282Y HFE has an extremely limited ability to form the HFE-TfR complex that limits Tf binding. The result is a dramatic increase in cellular iron entry [77]. The H63D variant, or H67D in mice, does not affect the translocation of HFE to the cell surface, and the association of HFE with TfR is minimally affected by the H63D mutation [11,73,77].

Both H63D and C282Y HFE have been associated with varying degrees of iron overload and resulting oxidative damage [40]. In humans, ~85% of HH patients are homozygous for C282Y HFE, and ~5% are compound heterozygous for C282Y and H63D HFE [41,72,81]. Homozygosity for C282Y HFE confers the highest risk for iron overload, but H63D HFE carriers are also at increased risk [82]. Furthermore, homozygosity for the H63D HFE variant is associated with increased serum transferrin saturation and ferritin levels, but most H63D HFE homozygotes have normal iron parameters [83]. Transferrin saturation has been shown to correspond to the HFE genotype in the following order from highest to lowest: C282Y/C282Y > C282Y/H63D > H63D/H63D > C282Y/+ > H63D/+ > +/+ [84]. With that being said, it is known that iron status is strongly influenced by sex, age, disease states, genetic factors, as well as environmental factors such as diet, alcohol consumption, and inflammation [10,11]. The role of each of these factors is not fully understood, let alone the complex interactions of multiple environmental and genetic factors working together. The clinical penetrance of HFE mutations is very low, and the presence of HFE variants is likely one of many factors that influences the heterogeneity of iron metabolism [41].

### 2.2. Prevalence of HFE Variants

The prevalence of HFE variants varies dramatically based on the population’s racial and ethnic background [85]. Within Caucasian populations, the H63D and C282Y HFE variants are estimated to be present in 15–30% and 2–5% of the population, respectively [11,85]. Regionally, the frequency of the H63D HFE variant is estimated to be 10–20% in the Americas and Europe (with some countries having a prevalence much higher than 20%), 1–5% in Asia, 1–10% in Africa, and less than 1% in Australia [41,42]. The C282Y HFE variant is fairly specific to Europeans and has a similar prevalence as HH, with a 1–5% prevalence in Europe and a frequency less than 1% in most non-Caucasian populations [41,42]. Interestingly, five separate studies have found that elite athletes carry HFE mutations nearly twice as frequently as ethnically matched control populations [86,87,88,89,90]. Furthermore, those with HFE mutations have a higher VO_2_ max than those with WT HFE, suggesting improved efficiency of oxygen delivery [87,88]. As H63D HFE causes systemically increased iron levels, it is possible that this leads to an increased rate of hemoglobin production for erythropoiesis and thus enhances oxygen delivery to the skeletal muscle to enhance athletic performance [88]. The increased prevalence of HFE mutations in athletes suggests a metabolic advantage in HFE variant carriers. This is in addition to the relatively high prevalence of this variant in the general population, suggesting a significant evolutionary benefit of HFE variants. Further studies into the athletic, metabolic, and neuroprotective consequences of HFE variants in different tissues can elucidate possible hormetic mechanisms related to oxygen delivery, oxidative stress, and antioxidant expression.

## 3. The Role of HFE Variants in Neurodegenerative Diseases

### 3.1. HFE Variants in Alzheimer’s Disease

Of the 21 studies that examined the effect of HFE variants in AD patients, the majority found a lack of association between HFE variants and AD risk or age of onset. However, it is important to consider the findings of studies that do show that HFE variants can modify AD given the immense heterogeneity of AD risk as well as the apparent low penetrance of these variants [41]. Table 1 outlines the findings of all studies that examined AD risk and prognosis in relation to the presence of HFE variants. Findings referring to the prevalence of HFE variants refer to those heterozygous and homozygous for the variant and do not refer to allele frequency unless otherwise noted. Comparisons with low statistical power or studies that lacked statistical analysis were not included. In studies with multiple cohorts, only the findings of combined cohorts were stated, unless otherwise noted. The S65C variant has been quantified in a few studies but never with a prevalence high enough to analyze statistically, so it has been excluded from discussion here.

Only one study found an overall increased prevalence of H63D HFE in AD patients, concluding that, after the ε4 variant of the Apolipoprotein E gene (*ApoE4*; the most common genetic variant in AD patients), H63D HFE is the variant with the strongest association with AD [91]. Other studies finding increased prevalence of H63D HFE in AD patients only reached significance in those also carrying *ApoE4* [92] or only in female *ApoE4* carriers, while finding a decreased prevalence of H63D HFE in male *ApoE4* AD patients [93]. The sex dependence observed in this study may be biologically significant given the sex differences in iron regulation [10]. It may also be important to note that these studies finding a higher prevalence of H63D HFE in AD patients were all from the US or Canada. A study from Spain, as well as a meta-analysis of 17 studies from many countries, found a lower prevalence of H63D HFE in AD patients [94,95]. It is possible that geography is an important factor in these conflicting findings, either due to different genetic backgrounds, environmental or dietary factors, or other unknown factors that affect iron metabolism in populations of different nationalities.

Several studies have found that the presence of H63D HFE affects the age of AD onset. Three studies of Caucasian and/or European cohorts found that AD patients with H63D HFE were more likely to have early-onset disease [43,96,97]. Other studies only found earlier AD onset in H63D HFE carriers that are homozygous for *ApoE4* [98] or only in male bi-carriers of H63D HFE and *ApoE4* [99]. These findings suggest a detrimental effect of H63D HFE in terms of AD risk, yet most studies find no association between H63D HFE and the risk of AD or altered age of onset.

C282Y HFE has also been cited as a disease modifier of AD in three studies. One study found a lower prevalence of C282Y in a Portuguese cohort of AD patients [96]. Two found an increased prevalence of C282Y HFE in AD patients that also carried the TF C2 variant [100,101]. These small studies do not provide conclusive evidence that C282Y HFE is a highly significant disease modifier, but the increased prevalence of AD in those who also carry TF C2, which is associated with increased redox-active iron levels, suggests that increased iron is a likely risk factor for AD. No studies have found that C282Y HFE affects the age of AD onset or modifies disease progression in any other manner. It is intriguing that C282Y HFE would be a less significant disease modifier than H63D HFE in AD. Under the assumption that neuropathology would be exacerbated with an increase in brain iron loading, C282Y HFE would be assumed to be the more detrimental variant in neurodegeneration. It is possible, given that the penetrance of C282Y HFE in HH is much higher than that of H63D HFE [82], that those with C282Y HFE frequently obtain treatment by phlebotomy mitigating brain iron overload risk or have other iron overload-related health risks that affect their representation in an age-related disease such as neurodegeneration. Both explanations likely play some role, and there may be other mechanisms of iron regulation yet to be understood affecting the role of HFE variants in AD.

**Table 1:** Comprehensive list of studies on the prevalence and effect of HFE variants in AD patients.

Study	Cohort Details	Prevalence of HFE Variants	Age of Onset	Other Findings
Yokoyama et al., 2015 [91]	185 AD patients (met IWG-2 criteria) and 283 healthy controls (scored ≥ 26 on MMSE or 0 on CDR Scale, no report of cognitive decline in the prior year and no known disease mutation) from the US.	Higher prevalence of H63D in AD patients compared to controls (OR = 2.83; *p* = 0.00164).	Not measured.	After *ApoE4*, H63D is the variant with the second strongest association with AD.
Pulliam et al., 2003 [92]	138 cognitively impaired patients (cognitive impairment and AD classification based on CERAD and NIA-RI criteria) and 67 healthy controls from the US.	Higher prevalence of H63D homozygosity and compound heterozygosity (H63D/C282Y) in *ApoE4* carriers with higher cognitive impairment (*p* = 0.03).	Not measured.	
Percy et al., 2008 [93]	54 sporadic AD patients (met NINCDS-ADRDA criteria for probable AD) and 58 healthy controls (matched for sex and age) from Canada.	Higher prevalence of bi-carriers of H63D and *ApoE4* in female AD patients compared to female controls (OR = 7.13, CI = 2.07–24.6; *p* = 0.003). Lower prevalence of H63D in male AD patients compared to male controls (OR = 0.198, CI = 0.055–0.715; *p* = 0.020).	Not measured.	
Blázquez et al., 2007 [94]	211 AD patients (met McKhann’s criteria) and 167 healthy controls from Spain.	Lower prevalence of H63D in AD patients compared to controls (18.0% vs. 29.9%; OR = 0.352, CI = 0.21–0.60; *p* < 0.05). No difference in prevalence of C282Y between AD patients and controls. This remained insignificant when stratifying by TF C2 presence (*p*-values not reported).	No difference between AD patients with or without H63D (*p*-value not reported).	
Lin et al., 2012 [95]	Meta analysis of 22 studies including 4365 AD patients and 8652 healthy controls genotyped C282Y and 17 studies including 2795 AD patients and 7424 healthy controls genotyped for H63D.	Lower prevalence of H63D in AD patients compared to controls (OR = 0.887, CI = 0.790–0.994; *p* = 0.037). No difference in prevalence of C282Y between AD patients and controls (OR = 1.039, CI = 0.914–1.181; *p* = 0.561).	Not measured.	
Correia et al., 2009 [96]	113 AD patients (met DSM-IV and NINCDS-ADRDA criteria for probable AD) and 82 healthy controls from Portugal.	Lower prevalence of C282Y in AD patients compared to controls (1.3% vs. 5.8%; *p* = 0.00197). No difference in prevalence of H63D between AD patients and controls (17.2% vs. 20.3%; *p* = n.s.).	Lower prevalence of H63D in AD patients with late onset (17.2% in controls vs. 16.2% in AD patients with onset after 66; *p* = 0.0504 vs. 11.5% in patients with onset after 75; *p* = 0.0125).	
Kauwe et al., 2010 [100]	1166 AD patients (met NINCDS-ADRDA criteria) and 1404 healthy controls (non-demented, matched for age and sex) from the US, UK and Canada.	No difference in prevalence of C282Y between AD patients and controls. Higher prevalence of bi-carriers of C282Y and TF C2 in AD patients compared to controls (SF = 2.4, CI = 1.38–4.19; *p* = 0.002).	Not measured.	
Robson et al., 2004 [101]	191 AD patients (met CERAD or NINCDS-ADRDA criteria for definite or probable AD), 69 MCI patients (defined by Petersen et al., 1999 [102]) and 269 healthy controls (without cognitive impairment and scored >80 on CAMCOG) from the UK.	Higher prevalence of bi-carriers of TF C2 and C282Y in female AD patients compared to female controls (OR = 7.0, CI = 1.5–33; *p* = 0.006). No difference in prevalence of H63D or C282Y between AD patients, MCI patients, and controls (H63D: 27.8% vs. 24.6% vs. 27.9%; *p* = n.s. C282Y: 15.7% vs. 24.6% vs. 11.9%; *p* = n.s.)	No difference between AD patients with or without H63D, C282Y, TF C2, or any combination of these variants.	
Alizadeh et al., 2009 [99]	268 AD patients (met NINCDS-ADRDA criteria) and 1970 healthy controls (no history of neurological disease) from The Netherlands.	No difference in prevalence of H63D or C282Y between AD patients and controls (*p*-values not reported).	Average 5.5 years earlier onset in men with *ApoE4* and H63D (73.2 ± 2.1 vs. 78.7 ± 1.6 years; *p* = 0.05).	
Sampietro et al., 2001 [43]	107 sporadic, late-onset AD patients (met NINCDS-ADRDA criteria) and 99 healthy controls (matched for age) from Italy.	No difference in prevalence of H63D between AD patients and controls (11% vs. 14%; *p* = 0.46). No difference in prevalence of C282Y between AD patients and controls (2% vs. 2%; *p* = 1.00).	Higher prevalence of H63D in AD patients with earlier onset (22% in patients with onset between 60–70 vs. 12% in patients with onset between 70–80 vs. 4% in patients with onset after 80; *p* = 0.004).	
Lehmann et al., 2012 [97]	1757 AD patients (met CERAD or NINCDS-ADRDA criteria for definite or probable AD), and 6294 healthy controls from multiple Caucasian cohorts.	No difference in prevalence of H63D between AD patients and controls (OR = 1.0, CI = 0.8–1.1; *p* = 0.6). No difference in prevalence of C282Y between AD patients and controls (OR = 1.0, CI = 0.8–1.2; *p* = 0.8).	Lower prevalence of H63D in AD patients with onset after 80 years (*p* = 0.02).	
Combarros et al., 2003 [98]	328 sporadic AD patients (met NINCDS-ADRDA criteria for probable AD).	Not measured.	No difference between AD patients with or without H63D (*p*-value not reported). Earlier onset in AD patients with H63D who are homozygous for *ApoE4* (5.1 years earlier than *ApoE4* heterozygotes; *p* = 0.014, and 6.1 years earlier than *ApoE4* non-carriers; *p* = 0.019).	
Berlin et al., 2004 [44]	213 sporadic AD patients (met NINCDS-ADRDA criteria for probable AD), 106 MCI patients (scored 0.5 on the CDR Scale) and 63 healthy controls from Canada.	No difference in prevalence of H63D between AD patients, MCI patients, and controls (33% vs. 26% vs. 34%; *p* = 0.43). No difference in prevalence of C282Y between AD patients, MCI patients and controls (5% vs. 6% vs. 10%; *p* = 0.33).	No difference between AD patients with or without H63D (*p* = 0.72). No difference between AD patients with or without C282Y (*p* = 0.93).	The presence of H63D or C282Y did not affect patients’ neuropsychological scores. No significant associations were found between *ApoE4* and either H63D or C282Y.
Avila-gomez et al., 2008 [103]	105 early-onset AD patients (containing the E280A variant of PSEN-1) and 220 healthy controls (non-demented and unrelated to study patients) from Colombia.	No difference in prevalence of H63D between AD patients and controls (32.4% vs. 29.57%; *p* = 0.8061).	No difference between AD patients with or without H63D (44.0 ± 5.1 vs. 44.42 ± 5.2 years; *p* = 0.7531).	
Candore et al., 2003 [104]	123 AD patients and 152 healthy controls (non-demented, unrelated to study patients) from Italy.	No difference in prevalence of H63D between AD patients and controls (13.8% vs. 11.2%; *p*-value not reported), remained insignificant when stratifying by ApoE status.	No difference between AD patients with or without H63D (67.79 ± 9.14 vs. 67.93 ± 2.54 years; *p*-value not reported).	C282Y and S65C were analyzed, but both had presence too low to analyze.
Corder and Beaumont 2007 [105]	90 possible AD patients, 80 probable AD patients, 145 definite AD patients (met NINCDS-ADRDA criteria for probable or possible AD or met CERAD criteria for definite AD; stratified by diagnosis) and 260 healthy controls (without cognitive dysfunction and scored >80 on CAMCOG) from the UK.	No difference in prevalence of H63D or C282Y among groups (H = 0.13 and 0.04, respectively).	Not measured.	
Giambattistelli et al., 2012 [106]	160 AD patients (met NINCDS-ADRDA criteria and scored ≤ 25 on MMSE) and 79 healthy controls (no evidence of conditions known to affect mental metabolism) from Italy.	No difference in prevalence of H63D between AD patients and controls (28.1% vs. 25.3%; *p* = 0.38).	No difference between AD patients with or without H63D (data not reported).	No difference in disease duration between AD patients with or without H63D (data not reported).
Lleo et al., 2002 [107]	108 AD patients (met NINCDS-ADRDA criteria for probable AD) and 110 healthy controls (no medical illness or cognitive impairment, unrelated to study patients) from Spain.	No difference in prevalence of H63D between AD patients and controls (42.6% vs. 34.5%; *p*-value not reported). No difference in prevalence of C282Y between AD patients and controls (3.7% vs. 3.6%; *p*-value not reported). Neither became significant when stratifying ApoE status or TF C2 presence (*p*-values not reported).	Not measured.	
Mariani et al., 2013 [108]	139 AD patients (met NINCDS-ADRDA criteria for probable AD and scored ≤25 on MMSE), 78 PD patients (met UK PDS Brain Bank criteria), 27 MCI patients (see paper for inclusion criteria) and 139 healthy controls from Italy.	No difference in prevalence of H63D between AD patients and controls (28.3% vs. 28.8%; *p* = 0.4). No difference in prevalence of C282Y between AD patients and controls (0% vs. 1.4%; *p* = 0.1).	Not measured.	
Guerreiro et al., 2006 [48]	130 AD patients (met DSM-IV and NINCDS-ADRDA criteria, had onset ≥ 65 and no family history of AD), 132 PD patients (met UK PDS Brain Bank criteria), 55 MCI patients (met criteria defined by Petersen et al., 1999 [102]), and 115 healthy controls (matched for age) from Portugal.	No difference in prevalence of H63D between AD patients and controls (33.6% vs. 35.6%; *p* = 0.76). No difference in prevalence of C282Y between AD patients and controls (4.6% vs. 4.3%; *p* = 0.92).	No difference between AD patients with or without either H63D (*p* = 0.93) or C282Y (*p* = 0.08).	
Vance et al., 2020 [109]	12,532 AD patients and 13,134 controls from the Alzheimer’s Disease Genetics Consortium (ADGC).	No difference in prevalence of C282Y between AD patients and controls (*p* = 0.40). This remained insignificant when stratifying by TF C2 presence (*p* = 0.23).	Not measured.	No detection of epistasis between TF C2 and C282Y (SF = 0.94; *p* = 0.48).

OR: odds ratio, CI: 95% confidence interval, n.s.: not significant, SF: synergy factor, MCI: mild cognitive impairment, TF C2: P570S mutation of transferrin, PSEN-1: presenilin-1, ApoE: apolipoprotein E, *ApoE4*: apolipoprotein E ε4 variant, DSM-IV: Diagnostic and Statistical Manual of Mental Disorders 4th edition, NINCDS-ADRDA: National Institute of Neurological and Communicative Disorders and Stroke and the Alzheimer’s Disease and Related Disorders Association, CDR: Clinical Dementia Rating, CERAD: Consortium to Establish a Registry for Alzheimer’s Disease, NIA-RI: National Institute of Aging-Reagan Institute, CAMCOG: Cambridge Cognition Examination, IWG-2: International Working Group 2, MMSE: mini-mental state examination, PDS: Parkinson’s Disease Society, ADGC: Alzheimer’s Disease Genetics Consortium.

### 3.2. HFE Variants in Parkinson’s Disease

In the field of PD research, it has long been hypothesized that the development of HH, regardless of HFE genotype, may result in excess brain iron deposition and contribute to the development of PD [110]. However, no association has been observed between HH and the risk of PD [111]. Similar findings have been found for HFE variants. Out of 13 studies to date that analyzed the effect of HFE variants in PD, most concluded that HFE variants do not modify disease risk. That being said, it is important to analyze the possible reasons for the significant results found in the minority of these studies to determine what role HFE variants may play in PD, given the broad context of geographic trends and heterogeneity of iron metabolism. Table 2 provides a comprehensive list of studies on the prevalence and disease-modifying effects of HFE variants in PD patients. In this table, prevalence refers to the homozygous and heterozygous presence of HFE variants and does not refer to allele frequency unless otherwise noted.

In PD, H63D HFE is not found in many studies to be a significant modifier of disease risk. Only one study found a higher allele frequency of H63D HFE in PD patients [47], and the two studies that investigated the effect of H63D HFE on the age of PD onset found no significant association [48,50]. However, a pivotal study in this debate found that H63D HFE protects against PD in agricultural workers exposed to paraquat (PQ), a toxin strongly linked to the risk of PD [53]. PQ exposure was associated with declining structural integrity in the SN; however, these pathologies were not observed in H63D HFE carriers exposed to PQ, highlighting possible neuroprotection in H63D HFE carriers [53]. PQ-exposed H63D HFE carriers also had higher serum iron levels than exposed workers with WT HFE, leaving the possibility that the observed neuroprotection is correlated to increased iron levels. It is compelling to consider that a hormetic mechanism may have influenced this outcome. Such a mechanism would dictate that increased iron from the H63D HFE gene variant leads to adaptation and heightened neuroprotection against oxidative insult, such as that caused by PQ-linked mitochondrial toxicity.

Broad analysis of the studies in Table 2 seems to show that the C282Y HFE variant is a more significant disease modifier than the H63D HFE variant in PD. Regarding the prevalence of C282Y HFE, one Australian cohort found a significantly lower prevalence in PD patients [112]. A meta-analysis of 8 studies confirmed a lower risk of PD in those with C282Y HFE [52]. While this finding may seem robust, there are two studies that found that C282Y HFE increases PD risk. A Portuguese study concluded that there is a higher prevalence of C282Y HFE in PD patients, while also finding it did not affect age of onset [48]. A large cohort study from The Netherlands found that only C282Y HFE homozygotes were at increased risk of PD and found a higher allele frequency of C282Y HFE in non-PD parkinsonism patients [51]. The geographical influence of these conflicting studies cannot be ruled out as a significant factor, given the immense biological implications of one’s nationality. The context of these findings in the broader field, where most studies find no association of PD risk and HFE variants, highlights the need to better understand the role of HFE variants in relation to other factors that affect iron metabolism to make more rigorous claims regarding mechanisms by which HFE variants may modify PD risk, onset, or progression.

**Table 2:** Comprehensive list of studies on the prevalence and effect of HFE variants in PD patients.

Study	Cohort Details	Prevalence of HFE Variants	Other Findings
Greco et al., 2011 [47]	181 sporadic PD patients (met UK PDS Brain Bank criteria) and 180 healthy controls from Italy.	Higher allele frequency of H63D in PD patients compared to controls (17.4% vs. 11.7%; *p* = 0.029). No difference in allele frequency of C282Y between PD patients and controls (0.8% vs. 0.8%; *p* = 0.995).	
Xia et al., 2015 [52]	Meta analysis of 8 studies including 1631 AD patients and 4548 healthy controls genotyped for C282Y and 7 studies including 1192 AD patients and 4065 healthy controls genotyped for H63D.	Lower prevalence of C282Y in PD patients compared to controls (11.47% vs. 12.03%; OR = 0.22, CI = 0.09–0.57; *p* = 0.002). No difference in prevalence of H63D between PD patients and controls (26.09% vs. 26.32%; OR = 0.99, CI = 0.84–1.17; *p* = 0.925).	
Buchanan et al., 2002 [112]	438 PD patients and 485 healthy controls (non-demented with no related illnesses; matched for age and sex) from Australia.	Lower prevalence of C282Y in PD patients compared to controls (10.7% vs. 16.5%; OR = 0.56, CI = 0.33–0.94; *p* = 0.027).	
Guerreiro et al., 2006 [48]	130 AD patients (met DSM-IV and NINCDS-ADRDA criteria, had onset ≥ 65 and no family history of AD), 132 PD patients (met UK PDS Brain Bank criteria), 55 MCI patients (met criteria defined by Petersen et al., 1999 [102]), and 115 healthy controls (matched for age) from Portugal.	Higher prevalence of C282Y in PD patients compared to controls (13.6% vs. 4.3%; *p* = 0.01). No difference in prevalence of H63D between PD patients and controls (32.6% vs. 35.6%; *p* = 0.47).	No difference in age of onset between PD patients with or without H63D (*p* = 0.98) or C282Y (*p* = 0.76).
Dekker et al., 2003 [51]	197 PD patients and 72 non-PD PS patients (from two cohorts; diagnosed according to the EUROPARKIN protocol) and 7983 healthy controls (from the Rotterdam study) from The Netherlands.	Higher prevalence of C282Y homozygosity in one cohort of PD patients compared to controls (1.5% vs. 0.3%; *p* = 0.03). No difference in prevalence of H63D between PD patients and controls (*p*-value not reported).	Higher allele frequency of C282Y in non-PD PS patients compared to controls (13.7% and 17.5% in PS cohorts vs. 6.1% in controls; *p* = 0.005 and *p* = 0.002, respectively).
Aamodt et al., 2007 [111]	388 PD patients (met criteria from Gelb et al., 1999 [113]), and 505 healthy controls (from Distante et al., 1999 [114]) from Norway.	No difference in prevalence of H63D between PD patients and controls (17.3% vs. 19.4%; *p*-value not reported). No difference in prevalence of C282Y between PD patients and controls (13.4% vs. 13.1%; *p*-value not reported).	
Borie et al., 2002 [46]	216 PD patients (demonstrated at least two of these signs: rigidity, bradykinesia, resting tremor, asymmetric onset; had improvement with levodopa treatment and absence of exclusion criteria), and 193 healthy controls (matched for age and sex) from France.	No difference in prevalence of H63D between PD patients and controls (36.3% vs. 33.9%; *p* = 1.000). No difference in prevalence of C282Y between PD patients and controls (7.0% vs. 8.8%; *p* = 0.7506).	
Akbas et al., 2006 [49]	278 idiopathic PD patients (met UK PDS Brain Bank criteria) and 280 healthy controls (no extrapyramidal disorders, matched for ethnicity) from Germany.	No difference in prevalence of H63D between PD patients and controls (16.0% vs. 14.1%; *p*-value not reported). No difference in prevalence of C282Y between PD patients and controls (4.7% vs. 5.8%; *p*-value not reported).	
Biasiotto et al., 2008 [50]	475 PD patients (met UK PDS Brain Bank criteria and demonstrated bradykinesia and at least one of these signs: resting tremor, rigidity and postural instability, positive response to dopaminergic therapy and absence of atypical features or other causes of parkinsonism) and 2351 healthy controls from Italy.	No difference in prevalence of H63D between PD patients and controls (14.53% vs. 13.3%; *p* = 0.31). No difference in prevalence of C282Y between PD patients and controls (1.7% vs. 1.6%; *p* = 0.88).	No difference in age of onset between PD patients with or without H63D (data not reported).
Mariani et al., 2013 [108]	139 AD patients (met NINCDS-ADRDA criteria for probable AD and scored ≤25 on MMSE), 78 PD patients (met UK PDS Brain Bank criteria), 27 MCI patients (see paper for inclusion criteria) and 139 healthy controls from Italy.	No difference in prevalence of H63D between PD patients and controls (31.2% vs. 28.8%; *p* = 0.5). No difference in prevalence of C282Y between PD patients and controls (4.1% vs. 1.4%; *p* = 0.2).	
Halling et al., 2008 [45]	79 idiopathic PD patients (demonstrated 2 of these signs: tremor, bradykinesia, rigidity) and 153 healthy controls (matched for age) from the Faroe Islands.	No difference in prevalence of H63D between PD patients and controls (29% vs. 25%; *p* = 0.60). No difference in prevalence of C282Y between PD patients and controls (13% vs. 14%; *p* = 0.50).	
Yi et al., 2023 [115]	Meta-analysis of 9 studies where HFE variants were genotyped containing 1801 PD patients and 4796 healthy controls.	No difference in prevalence of H63D between PD patients and controls (OR = 1.02, CI = 0.87–1.21; *p*-value not reported). No difference in prevalence of C282Y between PD patients and controls (OR = 0.88, CI = 0.72–1.08; *p*-value not reported).	
Saini et al., 2021 [116]	16,318 PD patients and 18,717 healthy controls from two cohorts (IPDGC and AMP-PD).	No difference in prevalence of H63D between PD patients and controls (17% vs. 16%; OR = 1.02, CI = 0.97–1.07; *p* = 0.53). No difference in prevalence of C282Y between PD patients and controls (5.2% vs. 5.9%; OR = 0.98, CI = 0.91–1.07; *p* = 0.69).	

OR: odds ratio, CI: 95% confidence interval, MCI: mild cognitive impairment, PDS: Parkinson’s Disease Society, NINCDS-ADRDA: National Institute of Neurological and Communicative Disorders and Stroke and the Alzheimer’s Disease and Related Disorders Association, MMSE: mini-mental state examination, PS: parkinsonism, DSM-IV: Diagnostic and Statistical Manual of Mental Disorders 4th edition, IPDGC: International Parkinson’s Disease Genomics Consortium, AMP-PD: Accelerating Medicines Partnership program for Parkinson’s Disease.

### 3.3. HFE Variants in ALS

Of the diseases discussed so far, HFE variants appear to play the most significant role in modifying ALS, with most studies finding some significant effect of HFE variants on ALS risk, age of onset, or disease duration. Table 3 contains a comprehensive list of studies on the prevalence and effects of HFE variants in ALS. In this table, prevalence refers to those heterozygous and homozygous for HFE variants and does not refer to allele frequency unless otherwise noted.

Several studies have found an increased prevalence of H63D HFE in ALS patients [54,55,56,58,62,117]. However, it is possible that the increased prevalence of H63D HFE in ALS patients is due less to this variant increasing the risk of disease but is, in fact, due to the increased disease duration found in ALS patients with H63D HFE [57,63,118], although one study only found this trend in ALS patients also carrying superoxide dismutase [Cu-Zn] (SOD1) mutations (one of the most common genetic mutations in ALS patients) [57]. In further support of H63D HFE being related to a protective milieu in ALS patients, two studies found that carriers of H63D HFE have later disease onset [55,61]. There are no studies that find H63D HFE has a lower prevalence in ALS patients or that it is related to earlier onset or faster disease duration. However, it is difficult to draw definitive conclusions given that several studies, including a meta-analysis, find no significant association between H63D HFE and ALS risk [60]. It is likely that a full understanding of the mechanisms by which H63D HFE affects ALS onset and progression will only become apparent when studied in the context of many additional risk factors for iron overload and neurodegeneration.

Regarding the C282Y HFE variant, the literature reports either a decreased prevalence of the variant in ALS patients [55,61,62] or no association with disease risk or progression [54,59]. C282Y HFE has not been extensively studied in ALS, but the majority of these studies conclude that this variant is protective against ALS. Whether this is due to hormetic adaptation to increased brain iron, decreased risk due to the likelihood of treatment of these patients with phlebotomy, or complex mechanisms yet to be described is still up for debate.

**Table 3:** Comprehensive list of studies on the prevalence and effect of HFE variants in ALS patients.

Study	Cohort Details	Prevalence of HFE Variants	Age of Onset	Other Findings
Sutedja et al., 2007 [55]	289 SALS patients (met El Escorial criteria for definite, probable, or possible ALS) and 5886 healthy controls from The Netherlands.	Higher prevalence of H63D homozygosity in ALS patients compared to controls (4.5% vs. 2.1%; OR = 2.2, CI = 1.1–4.1; *p* = 0.02). No difference in prevalence of H63D between ALS patients and controls (27.8% vs. 26.7%; OR = 1.1, CI = 0.8–1.4; *p* = 0.68). Trend toward lower prevalence of C282Y heterozygosity in ALS patients compared to controls (6.3% vs. 9.6%; OR = 0.6, CI = 0.4–1.0; *p* = 0.06).	Later onset in ALS patients with H63D compared to those without (62.9 vs. 58 years; *p* < 0.001). No difference between ALS patients with or without C282Y (56.6 vs. 59.6 years; *p* = 0.20).	No difference in disease duration between patients with or without H63D (2.6 vs. 3.1 years; *p* = 0.60). No difference in disease duration between patients with or without C282Y (3.1 vs. 3.0 years; *p* = 0.9). No difference in site of onset between ALS patients with or without either HFE variant (data not reported).
Goodall et al., 2005 [54]	166 SALS patients (met El Escorial criteria for definite or probable ALS) and 192 healthy controls (unrelated to study patients) from the UK and 213 SALS patients and 208 healthy controls from Ireland.	Higher prevalence of H63D in ALS patients compared to controls (34.3% vs. 22.0%; *p* < 0.001). No difference in prevalence of C282Y between ALS patients and controls (18.2% vs. 19.0%; *p* = 0.783).	No difference between ALS patients with or without H63D (56.14 ± 13.04 vs. 57.81 ± 12.18 years; *p* = 0.249).	No difference in site of onset between ALS patients with or without H63D (30.37% vs. 26.12% bulbar; *p* = 0.409).
He et al., 2011 [117]	195 SALS patients (met El Escorial criteria for definite or probable ALS) and 405 healthy controls (unrelated to study patients; matched for age, sex, and ethnicity) from China.	Higher prevalence of H63D in ALS patients compared to controls (10.26% vs. 3.21%; OR = 3.45, CI = 1.68–7.08; *p* < 0.001)	No difference between ALS patients with or without H63D (data not reported).	No difference in site of onset between ALS patients with or without H63D (data not reported).
Restagno et al., 2007 [56]	149 SALS patients (met El Escorial criteria for definite or probable ALS) and 168 healthy controls (matched for age, sex, and ethnicity) from Italy.	Higher prevalence of H63D in ALS patients compared to controls (28.8% vs. 14.8%; *p* = 0.004).	No difference between ALS patients with or without H63D (63.4 ± 9.3 vs. 60.2 ± 11.9 years; *p* = 0.002).	No difference in disease duration between ALS patients with or without H63D (783 vs. 993 days; *p*-value not reported). No difference in site of onset between ALS patients with or without H63D (23.26% vs. 24.53% bulbar; *p*-value not reported).
Wang et al., 2004 [58]	121 SALS patients (met El Escorial criteria for definite or probable ALS) and 133 healthy controls (normal neurological examination) from the US.	Higher prevalence of H63D in ALS patients compared to controls (29.75% vs. 14.29%; *p* = 0.0043)	No difference between ALS patients with or without either HFE variant (58.69 vs. 58.26 years; *p* = 0.8797).	No difference in disease duration between ALS patients with or without H63D (37.30 vs. 34.67 months; *p* = 0.6558).
Li et al., 2014 [62]	Meta-analysis of 6 cohorts containing 1692 ALS patients and 8359 healthy controls genotyped for C282Y and 14 cohorts containing 5849 ALS patients and 13,710 healthy controls genotyped for H63D.	Lower prevalence of C282Y in ALS patients compared to controls (8.98% vs. 10.89%; OR = 0.75, CI = 0.61–0.92; *p* = 0.006). Trend toward higher prevalence of H63D in ALS patients compared to controls (26.62% vs. 26.37%; OR = 1.14, CI = 0.98–1.33; *p* = 0.086).	Not measured.	
Praline et al., 2012 [61]	824 SALS patients (met El Escorial criteria for definite or probable ALS) and 447 healthy controls (matched for age, sex, and ethnicity) from France.	Lower prevalence of C282Y in ALS patients compared to controls (6.3% vs. 11.6%; OR = 1.95, CI = 1.3–2.92; *p* = 0.001). No difference in H63D prevalence between ALS patients and controls (27.0% vs. 30.2%; *p* = 0.29).	Later onset in ALS patients with H63D compared to without (61.3 ± 11.8 vs. 59.4 ± 12.9 years; *p* = 0.05). No difference between ALS patients with or without C282Y (60.7 ± 11.2 vs. 59.9 ± 12.8 years; *p* = 0.66).	No difference in disease duration between ALS patients with or without C282Y (*p* = 0.73). No difference in disease duration between ALS patients with or without H63D (*p* = 0.25). No difference in site of onset between ALS patients with or without either variant (H63D: 31.1% vs. 28.4% bulbar; *p* = 0.65, C282Y: 34.0% vs. 28.5% bulbar; *p* = 0.42). No difference in frequency of either variant between ALS patients with or without *ApoE4* (data not reported).
Yen et al., 2004 [59]	51 ALS patients (met El Escorial criteria for definite or probable ALS) and 47 healthy controls from the US.	No difference in prevalence of H63D between ALS patients and controls (13.7% vs. 11.7%; *p* = 0.831). No difference in prevalence of C282Y between ALS patients and controls (2.9% vs. 2.1%; *p* = 1.000).	No difference between ALS patients with or without either HFE variant (46.5 ± 12.8 vs. 49.8 ± 13.4 years; *p* = 0.425).	No difference in rate of progression (change of Appel score/months) in ALS patients with or without either HFE variant (1.52 ± 1.55 vs. 2.00 ± 2.43; *p* = 0.547).
Chiò et al., 2015 [57]	1119 ALS patients (definite, probable, probable-laboratory supported, and possible) and 1302 healthy controls (matched for age, sex, and ethnicity) from Italy and 232 ALS patients and 121 healthy controls from Sardinia (same criteria).	No difference in prevalence of H63D between Italian ALS patients and controls (28.2% vs. 27.2%; *p*-value not reported) or between Sardinian ALS patients and controls (33.6% vs. 34.7%; *p*-value not reported).	No difference between Italian ALS patients with homozygous, heterozygous, or without H63D (62.5 ± 11.2 vs. 62.2 ± 11.2 vs. 62.3 ± 11.2 years; *p* = 0.92) or between Sardinian ALS patients with homozygous, heterozygous, or without H63D (65.4 ± 10.3 vs. 60.6 ± 10.5 vs. 60.2 ± 12.8 years; *p* = 0.78).	No difference between Italian ALS patients with or without H63D (3.4 vs. 3.0 years; *p* = n.s.) or between Sardinian ALS patients with or without H63D (3.5 vs. 4.7 years; *p* = n.s.). Longer disease duration for Italian ALS patients carrying SOD1 mutations with H63D compared to without (15.3 vs. 2.1 years; *p* = 0.04). No difference in site of onset between ALS patients with or without H63D (26.3% vs. 26.9% bulbar; *p* = 0.91).
van Rheenen et al., 2013 [60]	3962 ALS patients and 5072 healthy controls (without any neuromuscular disease and unrelated to study patients) from The Netherlands, Belgium, Germany, Ireland, UK, Sweden, and Switzerland.	No difference in prevalence of H63D between ALS patients and controls (26% vs. 27%; *p* = 0.99).	No difference between ALS patients with or without H63D (homozygous *p* = 0.68, heterozygous *p* = 0.62).	No difference in disease duration between ALS patients with or without H63D (homozygous *p* = 0.51, heterozygous *p* = 0.26). This study also completed a meta-analysis of 6 previous studies, and their findings were consistent with their own study after adding these data.
Canosa et al., 2023 [118]	30 ALS patients from France and 153 ALS patients from Italy; all with SOD1 mutations.	Not measured.	No difference between ALS patients with or without H63D (*p* = 0.904).	Longer survival in ALS patients with H63D compared to those without (13.58 vs. 6.09 years; *p* = 0.034).
Su et al., 2013 [63]	38 SALS patients (met El Escorial criteria for definite, probable, or possible ALS) from the US (15 H63D homozygotes, 1 H63D heterozygote, and 22 WT HFE).	Not measured.	No difference between ALS patients with or without H63D (56.5 vs. 56.7 years; *p* = 0.973).	Average 28.1 months longer disease duration in ALS patients with H63D compared to without (75.3 ± 12.7 vs. 47.2 ± 11.0 months; *p* = 0.017). No difference in site of onset between ALS patients with or without H63D (12.5% vs. 13.6% bulbar; *p* = 1.000)

OR: odds ratio, CI: 95% confidence interval, n.s.: not significant, SALS: sporadic ALS, *ApoE4*: apolipoprotein ε4 variant, SOD1: superoxide dismutase 1.

### 3.4. Overall Effects of HFE Variants in Neurodegenerative Disease: A Possible Mechanism of Hormesis

HFE variants have been extensively investigated regarding their role in neurodegenerative disease due to the pathological effects of increased iron levels in the brain [119]. In cognitive impairment and dementia not associated with Alzheimer’s, Parkinson’s, or ALS, there are not many findings of increased risk due to HFE variants, but this may be due to a lack of investigation. In the few studies on HFE variants and risk of mild cognitive impairment (MCI), defined as impaired memory, language, or judgment that does not yet fit the definition of dementia, but is beyond that expected to occur as a result of aging, no studies of either HFE variant found an association with increased risk [108,120,121]. However, there are a few studies that may suggest that HFE variants affect one’s propensity for broader risk factors of dementia. C282Y HFE homozygosity has been associated with increased iron levels in the brain regions associated with the risk of dementia, such as the hippocampus and the thalamus [121]. It has also been reported that H63D HFE carriers may be at increased risk for white matter lesions, which are associated with a risk of cognitive decline [122]. Furthermore, it was found that H63D HFE homozygosity and compound heterozygosity (C282Y/H63D) were associated with a higher risk of dementia in males [123]. In females, only carriers of H63D HFE that also had diabetes had an increased risk of dementia, highlighting the importance of understanding the role of HFE variants in the context of disease states and other environmental factors [123].

The importance of studying the interactions between HFE variants and other risk factors for iron overload and neurodegeneration should not be understated. HFE variants are undoubtedly only one of many key factors that affect neurodegeneration pathology. Given the complex interaction of the long list of risk factors for neurodegenerative disease, it is entirely possible that H63D HFE affects neurodegeneration onset and progression through mechanisms that will only be understood when studied in combination with other key risk factors. In the age of large data sets and personalized medicine, biological insights can no longer be garnered from studies of simple associations but instead from rigorous analysis of many interacting risk factors. Meta-analysis will hence not simply distill the ‘correct answer’ to this question, but instead will dilute the importance of factors affecting some cohorts that may give important insight into mechanisms of iron metabolism that affect neurodegenerative pathology.

The lack of consistency in the literature may additionally be an important clue that the mechanism of HFE variants follows that of hormetic adaptation. Hormesis is a concept in toxicology defined as a biphasic dose-response relationship where high doses of a toxin exert the expected toxic effect, but doses below a toxin-dependent threshold allow for beneficial adaptation, leading to long-term protection against broadly-related stress exposure [68,69,70,71]. Studies elucidating the health benefits of exercise and fasting have found that transient increases in levels of reactive oxygen species (ROS) are key to improving health and preventing disease. Despite ROS having the reputation of a damaging stressor, these species are important signaling molecules that, when transiently elevated, lead to activation of antioxidant and cellular protection pathways, resulting in long-term protection against diseases related to oxidative stress such as heart disease, cancer, and neurodegeneration [70,71,124]. Additionally, many vegetable phytochemicals at high doses are carcinogenic or neurotoxic; however, at low doses, they have been repeatedly proven to prevent cancer and modify neuronal excitability to warrant long-term protection against excitotoxicity [125,126]. Given the support for hormetic mechanisms in disease prevention, it is possible that the increased iron and subsequent oxidative stress that accompany HFE variants fall within the ‘hormetic dose’ of stress that can decrease one’s propensity for developing neurodegenerative disease.

## 4. Animal and Cell Models of HFE Variants

Given the difficulty in controlling for genetic, dietary, and lifestyle factors in humans, mouse studies have formed the basis for understanding the effects of HFE variants by limiting the effects of these factors. Lab mice have less genetic variability than human cohorts, a controlled and consistent diet, and extremely similar lifestyles to one another. For this reason, the effects of HFE variants in mice are much easier to deduce than in human studies.

### 4.1. Rodent Models

The study of rodent models enables an interrogation of the mechanism by which HFE variants impact the brain. It has long been known that mutations that disrupt HFE function can produce HH in mice [127]. Mice with H67D or C294Y HFE, whether heterozygous or homozygous, also have increased redox-active iron concentrations in the liver and brain [11,64,128]. Mice homozygous for H67D HFE have increased H- and L-ferritin and decreased TfR, indicative of an adaptive increase in iron storage and decreased iron uptake [64]. H67D HFE mice also have increased astrogliosis and neuroinflammation, both markers of a stress response [64]. Mice with H67D HFE, either homozygous or heterozygous, have significantly increased oxidatively modified proteins in the brain at 6 months of age, but this is no longer increased at 12 months, suggesting an adaptive mechanism within this time frame [64]. Nrf2 was not significantly increased in mice with H67D HFE at 6 months but was increased at 12 months, highlighting the role of antioxidant expression as a significant component of the adaptation to iron-related stress in HFE variant brains [64]. These results suggest that there is an age-associated shift to a neuroprotective environment, possibly driven in part by upregulation of Nrf2 expression and activity [64]. The brains of H67D HFE mice have also been found to have decreased reduced glutathione (GSH) and oxidized glutathione (GSSG) levels, but increased GSH renewal capacity compared to WT mice [128]. This may suggest an increased antioxidant capacity. In fact, another study that supports an increased antioxidant capacity in H67D HFE mice showed they were protected from toxic manganese (Mn) deposition and consequent oxidative stress after olfactory Mn exposure [129].

H67D HFE mice have also shown protection against the development of Parkinsonian symptoms. WT mice injected with α-synuclein pre-formed fibrils (PFFs) show α-synuclein aggregation and loss of motor function. H67D HFE mice have less α-synuclein aggregation than WT mice and have no significant loss of motor function [66]. H67D HFE mice also show protection from the environmental toxin PQ, which is commonly used to model PD. When injected with PQ, H67D HFE mice had no increase in motor dysfunction as measured by the number of falls from a rotarod [67]. After WT mice were injected with PQ, they showed about a 50% decline in tyrosine hydroxylase (TH), a marker for dopaminergic neurons, compared to untreated mice. H67D mice interestingly have a baseline decrease in TH staining compared to WT, but when treated with PQ, there is no further decline [67]. This may suggest a difference in dopaminergic neuron development in H67D HFE mice, possibly including neuron death due to iron overload leading to the selection of neurons with stronger antioxidant defenses [67]. Additionally, WT mice injected with PQ had a 3-fold increase in microgliosis and a 4-fold increase in the number of microglia in the SN, but H67D HFE mice had a much smaller increase in these measures following PQ injection [67]. H67D HFE mice also had twice the L-ferritin expression, while WT mice had no change in expression after PQ injection, suggesting H67D HFE mice have adapted to store increased labile iron [67].

The only murine study of the H67D HFE that has not reported a protective response is an ALS model employing the G93A SOD1 mutation. Compared to mice with the G93A SOD1 mutation, double transgenic mice with H67D HFE and G93A SOD1 have shorter survival times and accelerated disease progression [130]. The double transgenic mice also had increased L-ferritin, microgliosis, caspase-3, and astrogliosis [130]. Interestingly, Nrf2 was decreased in the lumbar spinal cord of double transgenic mice compared to G93A SOD1 mice at the end stage [130]. This may seem contrary to the evidence of protection in H67D HFE mice; however, it may be explained by a previous study that found a shift from a stressed to a protected milieu that appears to occur between 6 and 12 months of age [64]. A diseased phenotype becomes evident by 3 months of age in G93A SOD1 mice, during which time the nervous system is likely still attempting to adapt to the stress caused by the HFE variant [130]. It is possible that the additional presence of the SOD1 mutation increases oxidative stress above the hormetic dose and thus does not allow hormetic adaptation to the HFE variant.

### 4.2. Primary Cell Culture Models

Primary cell culture models have been valuable in the study of HFE variants and have generally supported the hypothesis that H67D HFE promotes antioxidant defenses. After PQ exposure, WT primary astrocytes showed a larger decrease in viability than H67D astrocytes [65]. PQ treatment also reduced the mitochondrial mass in WT astrocytes, but there was a lesser reduction in H67D HFE astrocytes [65]. H67D HFE astrocytes showed heightened protection against mitochondrial membrane collapse caused by PQ and had increased Nrf2 and L-ferritin levels, signifying increased antioxidant response and iron storage to mitigate oxidative stress [65]. When Nrf2 was knocked down in H67D astrocytes, they were no longer significantly protected from PQ exposure and appeared similarly affected by PQ as WT astrocytes [65]. Additionally, H67D HFE astrocytes have higher baseline levels of NAD(P)H quinone dehydrogenase 1 (NQO1), a stress-inducible oxidoreductase and downstream target of Nrf2 [65]. Interestingly, H63D HFE carriers also have increased NQO1 levels in the CSF, based on PPMI clinical data [65]. Overall, this suggests Nrf2 is a key driver of adaptive protection against PQ in H67D HFE mice, and possibly humans as well. Based on the mechanism of antioxidant-driven adaptation, this mechanism likely protects against a broad scope of pro-oxidative insults.

### 4.3. Cell Line Models

Most cell line studies involving HFE variants focus on H63D HFE, but one study with HEK293T cells focuses on the impact of expressing C282Y HFE. It was found that these cells accumulated the mutant HFE protein in the ER and middle Golgi compartment where it underwent degradation [131]. C282Y HFE-expressing HEK293T cells also showed increased production and activation of interleukin-8, increased activation of the unfolded protein response (UPR) pathway, and increased cytochrome c levels [131].

SH-SY5Y cells transfected with HFE mRNA have been used to study the effects of HFE polymorphisms because these cells do not endogenously express HFE [132]. Interestingly, cell culture studies have generally shown that HFE variants lead to a stressed phenotype as opposed to the protected phenotypes described previously, likely because transfected cells have a heightened level of basal stress from transfection, and do not have sufficient time to adapt to stress incurred from the expression of HFE variants. SH-SY5Y cells transfected with H63D HFE have an increased labile iron pool, increased levels of protein carbonyls, and lower resting mitochondrial membrane potential compared to those transfected with WT HFE [66,132,133]. These metabolic consequences are common indicators of oxidative stress, which can occur from increased cellular iron levels [132]. In addition, H63D HFE-expressing SH-SY5Y cells grow slower than WT HFE-expressing SH-SY5Y cells [134]. It was also found that H63D HFE-expressing cells had significantly affected sphingolipid composition, which may affect cell signaling in a manner that supports neurodegenerative pathologies [134]. SH-SY5Y cells expressing H63D HFE also had increased cytochrome c in the cytosol, increased Bax in the mitochondria, and increased caspase-3 activity and subsequent apoptosis [133]. Increased tau expression and hyperphosphorylation are also common cellular pathologies of neurodegeneration, and H63D HFE-expressing SH-SY5Y cells have a dramatic increase in total tau levels and altered phosphorylation at several sites related to neurofibrillary tangle (NFT) formation. Further, the activity of GSK-3β, which is involved in tau hyperphosphorylation and NFT formation, is increased in SH-SY5Y cells expressing H63D HFE [135]. The expression of glutaminase, which catalyzes glutamine to glutamate in the regulation of excitatory signaling, is also increased in H63D HFE-expressing SH-SY5Y cells [136]. Furthermore, intracellular calcium levels, which tightly regulate glutamate signaling, are increased in H63D HFE-expressing cells [136]. Together, this suggests that H63D HFE in this cell line promotes a conducive milieu for glutamate excitotoxicity.

SH-SY5Y cells expressing H63D HFE have also been used to model the response to toxins related to neurodegeneration. Exposure of SH-SY5Y cells to β-amyloid led to increased cell stress and death, which was more evident in H63D HFE cells compared to those expressing WT HFE [133]. H63D HFE-expressing cells had a more dramatic induction of apoptosis after β-amyloid exposure than did WT HFE cells, suggesting greater vulnerability to β-amyloid toxicity [133]. In contrast, the PD-causing toxin PQ is less detrimental to H63D HFE SH-SY5Y cells than to WT HFE cells. After PQ exposure, WT HFE SH-SY5Y cells showed a larger decrease in viability than H63D HFE SHSY5Y cells. The WT cells also had a larger increase in ROS and a larger decrease in mitochondrial mass than the H63D HFE-expressing cells [65]. When treated with α-synuclein monomers or PFFs to induce α-synuclein toxicity, it was found that H63D HFE SH-SY5Y cells had less α-synuclein aggregation and less cell death than WT cells [66]. In H63D HFE SH-SY5Y cells, autophagic flux was increased compared to WT, possibly explaining decreased α-synuclein aggregation [66]. It is interesting that these cells expressing H63D HFE were protected from toxins specific to PD; overall, they showed a stressed phenotype. It is possible that the introduction of this foreign gene may increase stress above the hormetic threshold. Additionally, Nrf2 expression is inherently lower in neurons compared to astrocytes, which may partly explain the stressed phenotype in this cell line. Further, there may be complex implications for hybridizing a neuroblastoma cell line. Cancer phenotypes present in SH-SY5Y cells, particularly metabolic changes and increased mutation rate, may contribute to altered genetic expression, which may possibly explain a differential response to the addition of an aggregation-prone protein. Overall, these conflicting results point to the complex protective response (i.e., autophagy versus antioxidants) involved in hormetic adaptation to HFE variants and may provide keen insights into the impact of HFE variants on disease. For example, pathogenic processes that have a primary pathway involving autophagy (of misfolded proteins such as β-amyloid or α-synuclein) may be offered less protection from the HFE variant than those that have a primary pathogenic process of oxidative stress. However, it may be difficult to test this hypothesis, given the contribution of oxidative stress to protein oxidation and subsequent misfolding and aggregation. Additionally, protein aggregation has been found to promote both a pro-oxidative and anti-oxidative environment in different models [137]. Overall, the interplay of HFE variants in regulating oxidative stress and other cellular pathways is very complex, and more studies of these pathways in HFE mutant models are needed.

## 5. Mechanisms of Hormesis

The field of research aimed at elucidating the specific mechanisms of hormesis is still in its infancy, and studies of exercise-mediated hormesis are the most prevalent in the field. Exercise was shown to increase the concentrations of ROS and nitric oxide synthase (NOS) in the body, which activated NF-κB (nuclear factor kappa B), a transcription factor that enhances transcription of antioxidant genes as well as modulates inflammatory responses, cell growth, and apoptosis [71]. The most prominent changes induced by exercise included transcriptomic upregulation of antioxidant defenses (SOD and glutathione peroxidase) and increased mitochondrial metabolism (mitohormesis). The administration of an inhibitor of ROS production (such as antioxidant supplementation) mitigates exercise-induced adaptation [71,138]. A strong link has been proposed between elevated ROS production, induction of ROS defense, and increased mitochondrial respiration [124].

As described in Section 4, there is strong evidence for a mechanism involving the upregulation of Nrf2 activity in the H67D model. Nrf2 has also been found to be elevated in models of neurohormesis in response to H_2_O_2_ exposure or ischemic preconditioning [139,140]. Upregulated expression of Nrf2-driven genes leads to a pleiotropic cytoprotective defense, which has warranted neuroprotection in many models [141,142]. Nf2 activation by sulforaphane or tert-butylhydrozuinone (tBHQ) has been shown to protect neuronal cell lines against the oxidative insult initiated by dopamine, H_2_O_2_ and glutamate excitotoxicity [139]. Furthermore, Nrf2 is known to play a role in mitochondrial function and intermediary metabolism, yet the exact mechanisms are not fully understood [143]. Nrf2 knockdown decreases mitophagy, oxygen consumption rates, oxidative phosphorylation (OxPhos) efficiency, and mitochondrial membrane potential [144,145,146]. The upregulation of Nrf2 activates mitochondrial biogenesis and trafficking and increases metabolic substrate availability and ATP production [144,145,147]. Further research into the mechanisms by which mitochondrial function is improved following Nrf2 activation is still needed to understand the full signaling cascade.

A transiently increased concentration of ROS seems to be the common initiating event of hormesis, and the increased labile iron pool in the brains of H67D HFE mice is known to catalyze ROS production [148]. Hence, we propose that the hormetic mechanism in H67D HFE mice will demonstrate a similar pattern of heightened antioxidant defense (by increased transcription of genes regulated by NF-κB and Nrf2) and enhanced mitochondrial metabolism as observed in exercise-mediated hormetic mechanisms.

**Figure 2:**
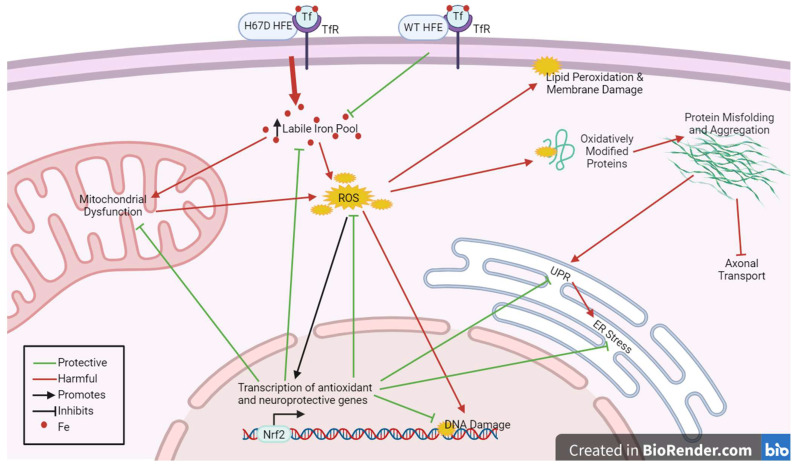
Our proposed mechanism of hormesis in the H67D HFE model.

Our proposal of the key mechanisms of hormesis in the H67D HFE model is outlined in Figure 2. We know that HFE serves to limit the labile iron pool, but the H67D variant leads to an increase in iron entry, which is known to increase ROS concentrations [148]. ROS can lead to oxidative damage such as DNA modification, lipid peroxidation, and protein carbonyl production (which has been observed in H67D mice). ROS also serve as signaling molecules that activate Nrf2- and NF-κB-driven transcription. Transcriptional alterations promote iron storage, inhibit oxidative damage and ER stress, and promote mitochondrial health and respiration.

## 6. Conclusions

We strongly believe the H63D HFE mutation has selective benefits, which have led to its persistence throughout evolution. One robustly demonstrated benefit of H63D HFE carriers is in oxygen delivery, likely explaining the dramatically increased prevalence of H63D HFE in elite athletes [86,87,88,89,90]. However, regarding the role of H63D HFE in modifying one’s propensity for or prognosis for neurodegenerative disease, one can draw no robust conclusions at this time. The key conclusion that can be drawn is that the variant is a significant contributor to disease heterogeneity, but the roles of innumerable genetic, environmental, and lifestyle factors that impact iron homeostasis and human disease cannot be disregarded. Our best method for mitigating genetic, environmental, and lifestyle variability is to study this variant in mice. The murine model clearly establishes that H67D HFE has a significant effect on brain iron metabolism, oxidative stress modulation, and subsequent protection from neurotoxic pathology. Given the similarities in iron homeostatic mechanisms between mice and humans, this effect is likely translatable, albeit more difficult to discern in humans. Thus, HFE mutations will certainly have an epidemiological impact on human disease processes, and we can discern this impact as we improve our clinical study design to include the important biological and lifestyle factors that impact iron levels and disease risk. We believe that the H63D/H67D variant increases brain iron levels to a concentration that falls within the hormetic dose for most organisms. Furthermore, most H63D/H67D organisms will adapt to this ‘subclinical iron overload’ (by the mechanism of hormesis proposed in Figure 2) and will be in a long-term state of heightened neuroprotection.

The concept of neurohormesis is still very much underrepresented in neuroscience research. There remains much to be known about the broad consequences and timeframes of hormesis, with the key difficulty stemming from the heterogeneity of the human population. As the medical field moves toward personalized therapeutic approaches, researchers need to expand their consideration of environmental and lifestyle factors that affect disease prognosis. Along these lines, many biological and pathological mechanisms could be elucidated if hormesis were better understood and applied in broader medical fields. Given the wide-ranging applications of hormesis in disease prevention, H63D HFE should be considered a modifier of any disease affected by iron overload and oxidative stress, namely neurodegenerative disease.

## Data Availability

No new data were created or analyzed in this study. Data sharing is not applicable to this article.
